# Neurosurgical Microvascular Anastomosis: Systematic Review of the Existing Simulators and Proposal of a New Training Classification System

**DOI:** 10.3390/brainsci14101031

**Published:** 2024-10-17

**Authors:** Lelio Guida, Martina Sebök, Marcelo Magaldi Oliveira, Christiaan Hendrik Bas van Niftrik, Fady T. Charbel, Marco Cenzato, Luca Regli, Giuseppe Esposito

**Affiliations:** 1 Department of Pediatric Neurosurgery, Assistance Pubilque Hôpitaux de Paris, Hôpital Necker Enfants Malades, Université de Paris Cité, 75015 Paris, France; lelio.guida@aphp.fr; 2 Departement of Neurosurgery, Clinical Neuroscience Center, University Hospital of Zurich, University of Zurich, 8091 Zurich, Switzerland; martina.seboek@usz.ch (M.S.); bas.vanniftrik@usz.ch (C.H.B.v.N.); luca.regli@usz.ch (L.R.); 3 Department of Surgery, School of Medicine, Federal University of Minas Gerais, Belo Horizonte 31270-901, MG, Brazil; mmagaldi@hotmail.com; 4 Department of Neurosurgery, University of Illinois at Chicago, Chicago, IL 60612, USA; fcharbel@uic.edu; 5 Department of Neurosurgery, Niguarda Great Metropolitan Hospital of Milan, 20162 Milan, Italy; marco.cenzato@ospedaleniguarda.it

**Keywords:** neurosurgical bypass, micro-anastomosis, microsurgical training, microvascular surgery

## Abstract

Background: The literature lacks a combined analysis of neurosurgical microvascular anastomosis training models. We performed a systematic literature search to provide an overview of the existing models and proposed a classification system based on the level of simulation and reproducibility of the microvascular anastomosis. Methods: The systematic literature search followed the PRISMA guidelines. We consulted MEDLINE, Web of Knowledge, and EMBASE independently for papers about bypass training models. Every training model was analyzed according to six tasks supposed to esteem their fidelity to the real operative setting by using a scoring system from zero to two. Finally, authors classified the models into five classes, from A to E, by summing the individual scores. Results: This study included 109 papers for analysis. Training models were grouped into synthetic tubes, ex vivo models (animal vessels, fresh human cadavers, human placentas) and in vivo simulators (live animals—rats, rabbits, pigs). By applying the proposed classification system, live animals and placentas obtained the highest scores, falling into class A (excellent simulators). Human cadavers and animal vessels (ex vivo) were categorized in class B (good simulators), followed by synthetic tubes (class C, reasonable simulators). Conclusions: The proposed classification system helps the neurosurgeon to analyze the available training models for microvascular anastomosis critically, and to choose the most appropriate one according to the skills they need to improve

## 1. Introduction

Cerebral bypass surgery has become a niche procedure that has to be performed in high-volume cerebrovascular reference centers by experienced and dedicated microvascular surgeons [1,2,3]. Maintaining sufficient know-how and improving technical skills represents one of the significant challenges for cerebrovascular training centers [4].

Adequate training in microsuturing and microvascular anastomosis is essential to develop the appropriate dexterity [5,6]. Ideally, training models should offer an adequate setting to train each step of this delicate procedure. Several training models have been proposed in the literature, which are traditionally grouped into ex vivo or in vivo models.

This paper analyzes, discusses, and classifies the main existing models used to practice microvascular anastomosis, based on the level of simulation and anastomosis reproducibility. To the authors’ knowledge, there is no such attempt in the literature so far.

## 2. Materials and Methods

### 2.1. Research Strategy

A comprehensive literature search was performed, following the PRISMA statement. MEDLINE, EMBASE and Web of Science were consulted between January 2019 and July 2023, using combinations of keywords and lexical variant about “training in microsurgical vascular anastomosis” ((education OR training OR simulator OR simulation) AND (anastomosis AND (microvascular) OR (microsurgical AND vascular) OR (microsurgery AND vascular)) OR ((micro-anastomosis AND vascular) OR (micro-anastomosis and vascular))). Since this review does not have a clear link to human health, it was not eligible for registration in PROSPERO.

All the original papers for training purposes on microvascular anastomoses written in English were included. Microsurgical anastomosis or micro-anastomosis refers to any vascular suture performed under an operative microscope, without a strict designation of minimum vessel size. Supermicrosurgical anastomosis is an anastomosis performed at 30–50× magnification on 0.3 to 0.8 mm vessels [7].

Exclusion criteria were papers about basic microsuture models (e.g., gazes), papers about microsurgical anastomosis of nerves, and micro-anastomosis papers not intended for training. Two independent reviewers (LG and MS) screened titles to find eligible papers; they read abstracts for primary evaluation of the studies and found additional studies in reference lists of relevant articles. No articles were retrieved from secondary sources.

### 2.2. Technical Classification of Brain Bypass Simulators: Six Tasks

The international board of experts in micro-neurovascular surgery defined 6 tasks during the international Bypass 2020 symposium in Zurich, Switzerland:

1. Vessel dissection: training with the dissection and the removal of adventitia.
2. Subarachnoidal dissection: training with the dissection of subarachnoidal spaces (cisterns).
3. Anastomosis variety: performing the standard micro-anastomoses within the same simulator (end-to-end, end-to-side, side-to-side).
4. Number of anastomoses/vessels/diameters of vessel: performing many microvascular anastomoses in the same simulator thanks to the availability of vessels of different diameters.
5. Stenosis-leaks: developing problem-solving strategies in case of anastomosis stenosis or anastomotic leaks (it requires perfusion and/or pressurization tests).
6. Thrombosis: developing problem-solving strategies in case of thrombosis of the micro-anastomosis.

By attributing the scores, authors considered all the simulators as perfused.

### 2.3. Scoring System

The authors applied to each simulator a scoring system ranging from zero to two:

- 0 points: the surgeon cannot execute the task by the simulator;
- 1 point: the task is described to be accomplished in an acceptable way;
- 2 points: the task is reported to be optimally doable by the simulator.

Agreement between the authors was ascertained by using Cohen’s K. Therefore, according to the total score, models were classified into five classes, from class A (excellent simulator) to class E (not suitable as a simulator) (Table 1).

## 3. Results

**Figure 1:**
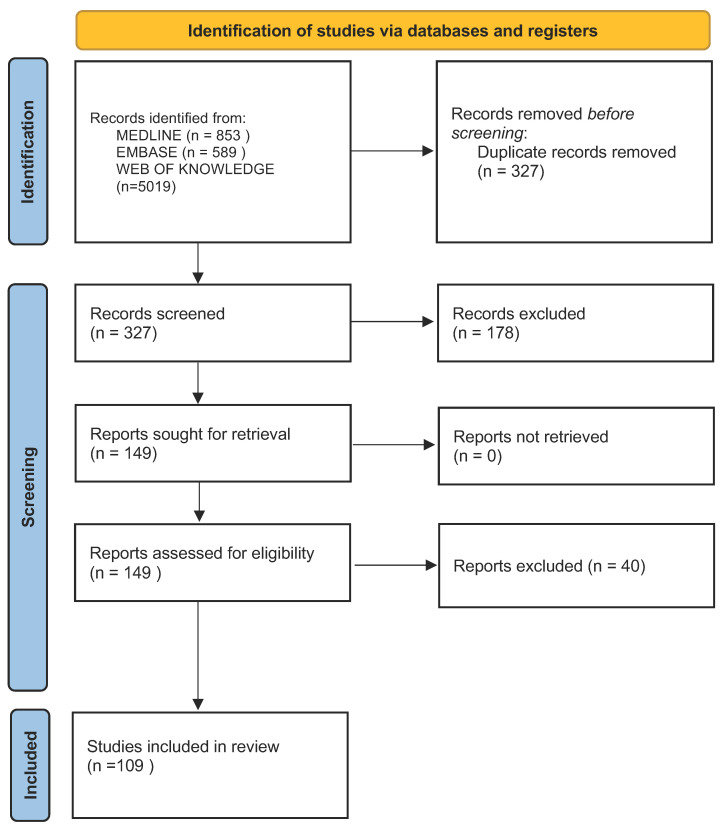
The PRISMA flowchart of the research strategy [8].

The literature search identified 327 non-duplicate records, of which 218 were excluded, and 109 articles were included in the analysis (Figure 1).

The models identified for analysis are described in the following sections.

### 3.1. Synthetic Tubes

Synthetic tubes have gained increasing success over the years because of their availability and low costs. Originally, manufacturers produced tubes in silicon [9], but current research proposes several alternative materials to better simulate the consistency of real arterial vessels (Gore-Tex, polyurethane, PVC, and PVA gelatin tubes).

In a recent blind randomized comparative survey between silicone and PVA tubes, the latter was demonstrated to better simulate rat vessels [10]. A three-layered synthetic vessel model has also been proposed [11].

Surgeons can train with single tubes, use practice cards composed of parallel tubes affixed to a pocketbook-sized card [12], or use commercialized supports [13]. Synthetic tubes offer the possibility to simulate standard types of vascular micro-anastomosis (task 3). The patency and strength of the anastomosis may be tested through the infusion of fluids [14] (task 5).

Disadvantages of these models include the impossibility to train with vessel and subarachnoid dissection (tasks 1 and 2) and to simulate thrombosis (task 6).

More complex and expensive models have been developed to overcome these limitations. Inoue proposed a model of an A3-A3 side-to-side bypass by putting two parallel prosthetic tubes on the bottom of a 6.5 cm deep paper box [15]. Takeuci presented an anatomic model using a mannequin head and small balloons filled with water, simulating the consistency of brain and Silastic tubes [16]. Ishikawa and Belykh developed skulls and brain models with craniotomies to simulate trans-Sylvian, subtemporal, suboccipital, and interhemispheric approaches [17,18]. Mori created an artificial skull and brain model to simulate posterior circulation revascularization [19]. In 2018, Cikla developed a skull and brain model with perfused artificial vessels to simulate both intra- and extracranial circulation and anastomoses [20]. Finally, the recently introduced virtual/augmented reality neurosurgical simulator boxes using synthetic perfused vessels in an anatomic accurate brain (e.g., Mycro model, Upsurgeon) represent an affordable and useful tool as an alternative to animal models [21].

### 3.2. Ex Vivo Training Models

#### 3.2.1. Animal Vessels (In Vitro)

Microvascular trainees can perform anastomoses on vessels harvested from animals sacrificed for other purposes. These materials are ideal because of their similarity to macroscopic and microscopic human vessels [22]. Arteries can be isolated and explanted or anastomosed in situ. Once the trainee completes the anastomosis, vessels can be perfused and pressurized [23] to assess the presence of leakages or stenosis (task 6). Chicken wings represent the most commonly used model, since they are widely available and affordable [24,25]. The chicken brachial artery is generally dissected from the surrounding connective tissues (task 1), cut, and sutured under a microscope. The harvesting of both brachial and radial arteries and their reimplantation has been suggested to simulate a superficial temporal artery–middle cerebral artery (STA-MCA) bypass [26]. Explanted animal vessels have been put deep into a grapefruit, between two slices [27], to simulate side-to-side distal anterior cerebral artery bypass. Other biological materials include the following:

- Cryogenically preserved rat [28] or human vessels (from tissues in lymph node dissection) [29,30];
- Porcine coronary arteries perfused through a pulsatile pump [31];
- Sections of turkey neck with both carotid arteries perfused [32];
- Turkey wings [33];
- Fresh chicken legs [34];
- Chicken feet [35];
- Oxen tongues [36];
- Pig spleen after splenectomy [37];
- Perfused porcine thighs [38];
- Bovine heart model [39];
- Rat cadavers with an extracorporeal perfusion device [40].

Turkey brachial arteries have been revealed to better approximate the real dimensions of human STA and MCA, and they were more appreciated than chicken wings in a survey among anastomosis course participants [41].

These models have several advantages: good reproducibility of a human artery’s physical characteristics (i.e., elasticity and consistency), accessibility [42], and conservation (most of the materials can be cheaply bought at supermarkets and stored over the long term in a freezer). A very recent comparative trial comparing non-living and living animal models found that trainees working with non-living chicken thigh models had better results than students training with living rats [43].

#### 3.2.2. Fresh Human Cadavers

As shown by Sindhu et al. in a randomized controlled trial, the higher the model fidelity to real-life experience in the operating room, the faster the acquisition of the required skill [44].

Several different cadaveric models have been developed. Entirely pressurized fresh cadavers are useful to simulate high-flow conventional carotid-to-middle cerebral artery bypass with interposition of a previously harvested radial artery graft [45]. Cadaveric heads, whose arteries and veins have been cannulated and connected to a closed pumping circuit with subarachnoid cisternal tubes, can simulate the cisternal circulation of blood and CSF [46,47]. Latex-injected non-pressurized cadaveric heads are used to perform the main types of cerebral anastomoses after the removal of superficial intravascular latex [48]. Perfused and fixed human cadaveric brain [49] has been enclosed with white silk clothing (simulating the dura) and inserted into human skulls [50].

Despite the high anatomical fidelity, these models are expensive; they are not easily accessible and do not offer a large number of anastomoses to perform. These disadvantages restrict their use to specific purposes for advanced training or research (for instance, assessing the intracranial feasibility of new intracranial bypass techniques).

#### 3.2.3. Placenta

Human and bovine placentas represent effective models for microsurgical training [51,52,53]. According to the histological composition of its vessels, the human placenta may be considered an appropriate model for STA-MCA anastomosis. In contrast, the thicker and larger diameter of bovine placenta is a good model for internal carotid artery–radial artery–middle cerebral artery (ICA-RA-MCA) bypass [54].

Recently, Magaldi Oliveira systematically defined the nomenclature of placenta vessels and correlated them with major intracranial arteries [51]. When perfused and pressurized, placental arteries simulate intracranial vessels, and the trainee can perform a great variety of simulation scenarios in a single model, allowing for many repetitive exercises and surgical error exploration. With a single placenta model, the trainee can perform a lot of micro-anastomoses and other procedures such vessel graft reimplantation/transplantation. A recently introduced pulsatile flow system can further improve the surgical fidelity of these models [55] (tasks 4). A placenta can be inserted into a skull model [54] to increase the anatomical fidelity. Trainees can check bypass vessel patency with indocyanine green light microscopic vision (task 5) or analyze it with the application of the anastomosis lapse index (ALI), based on the number of average errors per anastomosis performed [56]. Human [57] and bovine [54] placentas are usually affordable and obtainable, being a waste product of birth and calving. Placenta models are among the most promising tools available for training in microvascular anastomoses, due to their similarity to cerebral vessels, accessibility, and affordability.

### 3.3. In Vivo Training Models (Live Animals)

Microsurgical training based on live animals has been the paradigm of microsurgical training for years, and it currently remains a very accurate, practical, and appreciated method to simulate microvascular anastomoses [58]. In vivo models offer the unique advantage of reproducing physiologic heart pulsation and coagulation. These models allow one to simulate and manage acute intraprocedural complications such as intraluminal thrombosis (task 6). Rats are most commonly used because of their availability, uncomplicated perioperative care, and limited costs (about USD 120/animal [59]). Several experimental models have been proposed over the years:

- Side-to-side anastomosis involving a bilateral common carotid artery [60], portal vein and vena cava [61], femoral vein and artery [62], bilateral common iliac arteries [58], and internal and external carotid artery [60];
- End-to-end anastomosis involving the common carotid [58], abdominal aorta [63], femoral artery [64], and external jugular vein [65];
- End-to-side anastomosis involving the bilateral common carotid artery (“half-ring model”) [66], common carotid artery and external jugular vein [66], superficial caudal epigastric artery and contralateral femoral artery [66], iliolumbar vein and inferior vena cava [67], left and right common iliac arteries [58,68], middle sacral artery and common iliac artery [68].

Most animals are euthanized after the surgery: in these cases, the quality of anastomosis is assessed by harvesting the artery from the living animal or during the autopsy.

Contrarily, some authors have reported on the long-term patency and onset of late complications after training, for example, in the side-to-side internal and external carotid artery bypass models and in the side-to-side femoral artery and vein anastomosis model [69] (task 6).

Two further proposed models involve rats. The multiple lymphatic–venous anastomosis (MVLA) model consists of the anastomosis of lymphatic vessels into the lumbar vein [70]; the rat tail revascularization model offers the advantage of assessing the functionality of the anastomosis by evaluating its vitality [71].

Even if in vivo models cannot reproduce surgical anatomy, they have unique advantages in terms of high-fidelity simulation of almost all surgical steps, including the occurrence and management of intra-operative (anastomosis leak, acute thrombosis, vasospasm, or vascular stenosis) and postoperative complications (delayed thrombosis of the anastomosis, and formation of pseudoaneurysms) (tasks 5–6) [72]. These are the reasons why living rats are still among the commonly used and appreciated training models worldwide [73].

However, the availability of these models progressively reduces because of ethical reasons and the high costs of managing an animal laboratory. A recently published systematic review highlighted the lack of evidence regarding the need to teach microsurgical or microvascular skills [74].

### 3.4. Classification

There was a strong agreement between authors (92%), with a Cohen K of 0.9. Based on the proposed scoring system (Table 1), the simulation devices are classified as follows (Table 2):

1—Synthetic tubes—Grade C—reasonable simulators: They allow one to perform all the types of micro-anastomoses (task 3) and to perform several anastomoses (task 4). More sophisticated models using synthetic tubes allow for the evaluation of the immediate patency and strength of the anastomosis (task 5) [14]. Conversely, tubes are not suitable for training surgeons in vessel or subarachnoid microdissection (task 1 and 2), or to simulate the onset of thrombosis (tasks 7).

2.1—Animal vessels (ex vivo)—Grade B—good simulators: They allow one to train with all the micro-anastomoses (task 3) several times thanks to their availability (task 4), with the advantage of improving some abilities in vessel microdissection (adventitia removal) (task 1), even if the experience of dissecting a vessel from muscles and fat does not perfectly fit subarachnoid dissection. When perfused/pressurized, they mimic human vessel pulsation, allowing the surgeon to assess the presence of stenosis and leaks [23,75] (task 5). However, these models are not suitable to simulate thrombosis (task 6).

2.2—Fresh human cadavers—Grade B—good simulators: Fresh human cadavers allow one to train some abilities with vessel microdissection (task 1) and to perform all the standard micro-anastomoses (task 3). This model optimally allows one to train in arachnoid dissection (task 2). Perfusion and pressurization of the model allow for the evaluation of stenosis or leaks of the micro-anastomosis (task 5). Trainees can perform micro-anastomosis with vessels of different diameters and depths (task 4). Cadavers models are limited by the impossibility to reproduce thrombosis (task 6).

2.3—Placenta—Grade A—excellent simulator: Placenta models allow the optimal achievement of all the tasks, with the exception of simulating thrombosis scenarios (task 6). The big advantage is that it is possible to perform the tasks many times as needed, in vessels of different sizes (task 4) [51]. Dissecting placenta vessels simulates arachnoidal dissection.

3—Live animals (in vivo)—Grade A—excellent simulators: Given the presence of an active coagulation system, these are the only simulators that develop thrombotic complications (task 6). These models offer a unique surgical experience when facing challenging complications such as bleeding, micro-anastomotic leaks, and thrombosis. However, live animals are not optimal to repeat the exercise as many times as needed and allow limited training in subarachnoid dissection. In selected protocols, a post-anastomotic follow-up period can be applied to evaluate the delayed bypass patency [67]. Moreover, ethical concerns exist regarding the use of live animals.

## 4. Discussion

**Figure 2:**
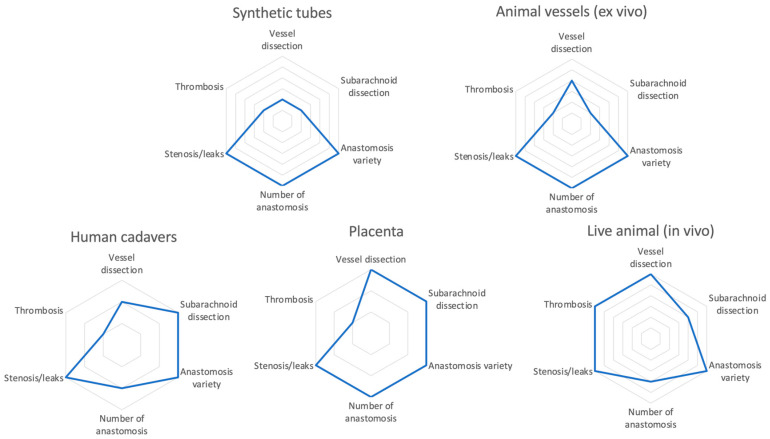
Spider graphs presenting the features of the training models. In clockwise sense: basic microsuture models, synthetic tubes, animal vessels (in vitro), human cadavers, placenta, and live animals (in vivo).

The proposed classification system provides a valuable resource to the microvascular surgeon to critically analyze the multiplicity of available devices and choose the most appropriate model for cerebral bypass surgery according to the skills they need to improve (Figure 2). To the authors’ knowledge, this is the first paper to propose a classification system of the training models.

A limitation of this classification is that it only takes into account the technical aspects of performing a micro-anastomosis and considers neither costs nor availability nor the ethical issues of the models. This is mostly true for class A and B models that allow for achieving the highest number of skills. Placenta has strong overall availability and cheaper direct and indirect costs in comparison to human cadavers and in vivo models. In this sense, placenta models represent valid simulators for every trainee (mostly intermediate and advanced), especially in reduced-budget educational programs. Placenta also represent a very good model for experienced surgeons who want to regularly exercise their skills or try/master new techniques.

A significant advantage of this classification is its dynamic nature. It can be continually updated if researchers develop new educational models ameliorate existing models.

Importantly, the highest scored models (grades A and B) do not represent the best simulators for all trainees. Mokhtari highlighted that entry-level surgeons who had previously undergone training with Silastic tubes performed significantly better in terms of anastomotic quality and average time when working with live animals [63].

In 2016, the analysis of the 10-year training results in an Okayama research center revealed that initially performing microsurgery on silicon tubes and chicken wings enabled a reduction in the number of animals required later in the training program [76].

An analysis by Pafitanis, involving novices training with coupler-assisted microvascular anastomoses of a three-layer silicon vessel, objectively assessed the learning curve of the trainees. This analysis proposed objective thresholds for ethical animal model training and safe, supervised clinical sessions in the operating theater. These studies support the idea that the educational process of the cerebral bypass surgeon requires stepwise training.

Based on this classification, we suggest that entry-level trainees first master the micro-anastomosis techniques on class C simulators. Thereafter, intermediate and advanced trainees could use placenta to master microdissection and micro-anastomoses techniques. If needed, advanced trainees may consider the use of fresh cadavers with high fidelity of the brain and angioanatomy, which offer the unique advantage of simulating EC-IC or IC-IC bypasses with or without interposition of a graft, or live animals, for working with real rheological properties.

In this sense, this scoring system can support the development of educational programs in neurovascular micro-neurosurgery, helping to select the most adapted training models according to trainee level.

Finally, despite the notable impact of training on the learning curve of cerebrovascular microsurgeons, it is mandatory to emphasize the importance of working alongside expert cerebral bypass surgeons [1,77].

## 5. Conclusions

The proposed classification considers the most relevant skills required by a neurosurgeon to perform a microvascular anastomosis. This system helps trainees to critically analyze the available models and to choose the most appropriate according to their level (entry-level vs. intermediate vs. advanced) and the skills to be improved. This way, it may also further reduce the use of live animals as training models.

## Figures and Tables

**Table 1:** Features of the classification system.

Tasks		Score	Sum	Grade
1	Vessel dissection	2 task optimally doable	10–12	A (excellent simulator)
2	Subarachnoidal dissection		7–9	B (good simulator)
3	Variety of the anastomosis	1 task doable but not in a proper way	4–6	C (reasonable simulator)
4	Number anastomosis/vessel/diameter			
5	Stenosis leaks	0 task not doable	1–3	D (poor simulator)
6	Thrombosis		0	E (not a simulator)

**Table 2:** By using this scoring system, models have been classified into 5 classes, from class A (excellent simulator) to class E (not suitable as simulator): see Table 1.

Task	Synthetic Tubes	Animal Vessels (Ex Vivo)	Human Cadavers	Placenta	Live Animals (In Vivo)
Vessel dissection	0	1	1	2	2
Subarachnoid dissection	0	0	2	2	1
Variety of the anastomosis	2	2	2	2	2
Number anastomosis/vessel/diameter	2	2	1	2	1
Stenosis leaks	2	2	2	2	2
Thrombosis	0	0	0	0	2
TOTAL OF POINTS (class of the simulator)	6 (C)	7 (B)	8 (B)	10 (A)	10 (A)
